# Free-energy landscapes of membrane co-translocational protein unfolding

**DOI:** 10.1038/s42003-020-0841-4

**Published:** 2020-04-03

**Authors:** Christian Bech Rosen, Hagan Bayley, David Rodriguez-Larrea

**Affiliations:** 1 Department of Chemistry, University of Oxford, 12 Mansfield Road, Oxford, OX1 3TA UK; 2 Biofisika Institute (CSIC, UPV/EHU) and Department of Biochemistry and Molecular Biology (UPV/EHU) Barrio Sarriena s/n, Leioa, 48940 Spain; 3 Present Address: Novozymes A/S, Biologiens Vej 2, 2800 Kgs. Lyngby, Denmark

**Keywords:** Single-molecule biophysics, Molecular biophysics

## Abstract

Protein post-translational translocation is found at the plasma membrane of prokaryotes and protein import into organellae. Translocon structures are becoming available, however the dynamics of proteins during membrane translocation remain largely obscure. Here we study, at the single-molecule level, the folding landscape of a model protein while forced to translocate a transmembrane pore. We use a DNA tag to drive the protein into the α-hemolysin pore under a quantifiable force produced by an applied electric potential. Using a voltage-quench approach we find that the protein fluctuates between the native state and an intermediate in the translocation process at estimated forces as low as 1.9 pN. The fluctuation kinetics provide the free energy landscape as a function of force. We show that our stable, ≈15 k_B_T, substrate can be unfolded and translocated with physiological membrane potentials and that selective divalent cation binding may have a profound effect on the translocation kinetics.

## Abstract (web summary)

Rosen et al showed, at the single-molecule level, that a protein fluctuates between the native and an intermediate state during its forced translocation through a transmembrane pore, and the kinetics of fluctuations provide the free energy landscape as a function of force. They show further that apparently minor events, like the binding of a divalent metal ion, can have major impacts on the translocation kinetics.

## Introduction

After protein synthesis in the cytosol many proteins must translocate across a membrane in order to reach their final compartment or the extracellular medium. Among them we find mitochondrial protein precursors^1^ and many secretory preproteins translocated into the endoplasmic reticulum (ER) in eukaryotes^2–5^ or the periplasm in prokaryotes^6–8^. Frequently, unfolded forms of proteins are subject to posttranslational translocation^9^ through a 1–2 nanometer wide protein pore present in the lipid bilayer^5,10,11^. Several multi-component systems mediate the unfolding and translocation of protein substrates^12^, for example the SecYEG channel in translocation to the periplasm of prokaryotes, the Sec61αβγ channel in translocation to the ER of eukaryotes^5,13,14^, and TIM-TOM in translocation to the mitochondrial matrix^15^. The post-translational translocation of substrates in these systems includes^5,6^ (i) targeting the protein substrate to the translocon; (ii) substrate unfolding; and (iii) translocation of the unfolded polypeptide chain through the translocon. While in some cases the process may require accessory proteins, such as chaperonins^2,16,17^, and the input of energy mediated by ATP, in others the presence of electrochemical gradients (approximately −180 mV in the mitochondrion)^18^, a membrane translocon, and a short, positively-charged targeting sequence at the N terminus of the substrate is enough for efficient targeting, unfolding and translocation^4,19–21^.

Once a protein substrate is placed at the pore entrance, unfolding proceeds mechanically. The force generated by the membrane potential on the N terminus is estimated to be in the range from 6 to 16 pN^22^ a force achievable with single-molecule approaches used to study co-translocational protein unfolding^19,21,23–29^. Meanwhile protein refolding during translocation has remained elusive to experimentation, in particular with single-molecule approaches. Protein refolding is important because it can generate a mechanical force that prevents translocation if occurs in the donor compartment or promote chain release from the translocon if occurs in the recipient compartment.

Here we develop a voltage-quench approach that allows observation of the dynamics of a thioredoxin substrate as it is pulled through the α-hemolysin pore at voltages as low as +30 mV, exploring a force-regime comparable to that found in mitochondria and other important compartments such as the endoplasmic reticulum and the plasma membrane. By this means, we observe folding-unfolding transitions of single thioredoxin molecules, providing free energy measurements and the folding and unfolding kinetics. The unfolding free energy change is similar to that obtained by other means, but the kinetics depend strongly on the applied voltage, and unfolding is promoted with forces as low as ≈2.5 pN. Importantly, we show how apparently minor events such as the binding of a single divalent metal ion can have major effects on the translocation kinetics. Our general model provides a framework with which to understand the energetics of additional protein posttranslational translocation systems.

## Results

### Co-translocational unfolding at low applied potentials

**Fig. 1:**
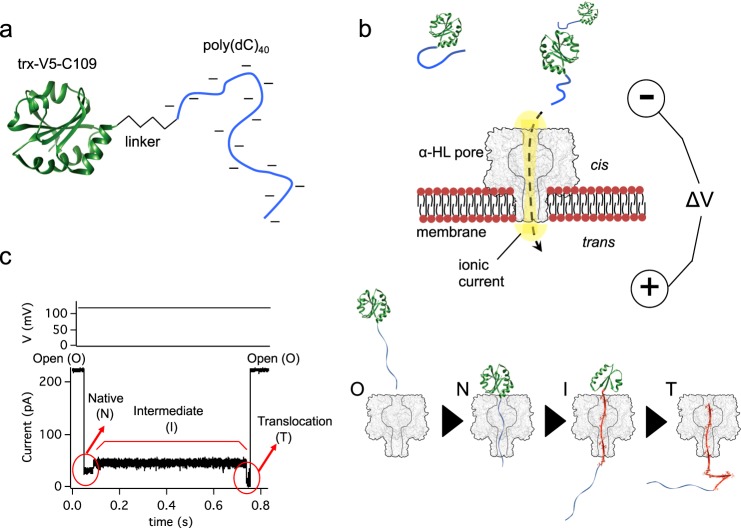
Co-translocational unfolding mediated by a transmembrane pore. **a** A disulfide-free mutant of thioredoxin with 3 mutations that increase its stability^31^ (A22P-I23V-P68A) was modified at a C-terminal cysteine with a 5′-maleimide-poly(dC)_40_ to produce the construct Trx-V5-C109-poly(dC)_40_. **b** A single α-hemolysin pore (α-HL) inserted in a lipid bilayer conducts an ionic current in response to an applied electrical potential and the negatively charged oligonucleotide is pulled into the pore. **c** At a constant potential of +120 mV, co-translocational unfolding occurs as manifested in the current trace, which reflects the molecular steps in the process: O, open pore; N, folded protein with the oligonucleotide threaded into the pore; I, an intermediate in the unfolding process with the C terminus threaded into the pore (the unfolded polypeptide segment is in red); T, translocation of the unfolded protein.

We examined protein co-translocational unfolding with a disulfide-free (C32S-C35S) stabilized (A22P-I23V-P68A) mutant of thioredoxin carrying a poly(dC)_40_ oligonucleotide covalently linked to a C-terminal cysteine by a maleimide group placed at the 5′ end of the oligonucleotide^25,30^ (V5-C109, Fig. 1a). Our single-molecule experiments were carried out by monitoring the ionic current passing through a single α-hemolysin (α-HL) pore inserted into a lipid bilayer that separated two buffer-filled compartments^31^ (Fig. 1b). We used the α-HL pore because the ionic current that passes through it is very sensitive to threaded polymer sequences, allowing both RNA and DNA sequencing^32–35^ and the identification of protein modifications^36^. Also, by using this approach, the V5-C109 co-translocational unfolding pathway has already been finely characterized^25,27^ (Fig. 1c). When a positive potential was applied, the oligo was driven into the pore from the cis side (Fig. 1b) and the electrophoretic force initiated the unfolding and translocation of the attached protein through an intermediate in which the C-terminal part of the protein was unfolded and threaded into the pore^25^. During the initial threading of the oligonucleotide into the pore at +120 mV, when the protein was in the native state, a characteristic low-conductance level was observed with I_res,%_ = 15.1 ± 0.5% [mean ± standard deviation (SD)], where is I_res,%_ is the I_res_ expressed as a percentage of the open pore current. The native state was transformed into a partly unfolded, still threaded intermediate at a rate of 50 ± 11 s^−1^ [rates are given hereafter as mean ± 95% confidence interval (CI) in the parameter estimation, see Methods section] (Supplementary Fig. 1). The intermediate state (I) had a higher ionic conductance than the structure (N) containing the native state (I_res,%_ = 21.1 ± 0.5%, [mean ± SD]). The sensitivity of this I_res_ to mutations and/or post-translational modifications, together with the lack of voltage dependence on further unfolding of I suggest that at this stage the intermediate fully threads the pore^25,36^. Finally, the intermediate completes unfolding with a rate of *k*_I→T_ = 2.8 ± 0.5 s^−1^ (Supplementary Fig. 1) and the unfolded protein (T) escaped from the pore restoring the open pore current (Fig. 1c).

**Fig. 2:**
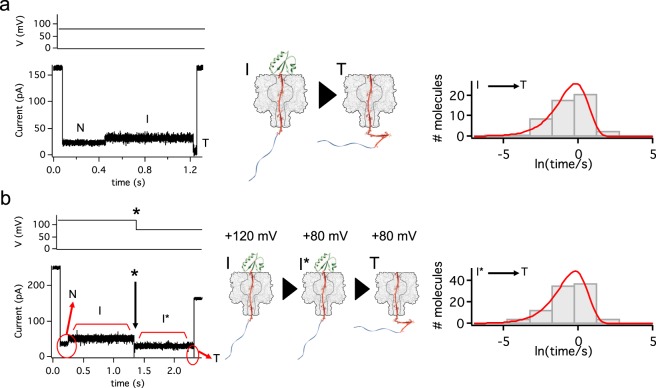
Co-translocational unfolding is memoryless. **a** At a constant voltage of +80 mV (upper left panel), V5-C109-poly(dC)_40_ was driven into the αHL pore. The intermediate (I) unfolded and was translocated through the pore (T). The lifetimes of I (*n* = 53) were fitted to an exponential distribution, yielding *k*_I→T_ = 1.1 s^−1^ ± 0.1 s^−1^. **b** With the same α-HL pore used in a), V5-C109-poly(dC)_40_ was captured at a higher potential (+120 mV). When I appeared the potential was reduced to +80 mV (asterisk). The lifetimes of I* after the step to +80 mV (*n* = 98) were fitted to an exponential distribution, yielding *k*_I*→T_ = 1.05 ± 0.04 s^−1^.

The lifetimes of the intermediate ‘I’ were distributed exponentially at +120 mV (Supplementary Fig. 1). In order to confirm that the further unfolding of ‘I’ is a memoryless process (i.e., the unfolding time does not depend on how much time has elapsed already, or the previous conditions) we carried out experiments using the same α-HL pore throughout because different pores (i.e., different experimental setups) may give slightly different rates. We studied the lifetimes of I, at a constant potential of +80 mV, and obtained exponentially distributed dwell times equivalent to an unfolding rate of *k*_I→T_ = 1.09 ± 0.08 s^−1^ (*n* = 53; Fig. 2a). Using the same pore, the lifetime distribution could be obtained after a voltage quench, applying first +120 mV until ‘I’ appeared, and then dropping the potential to +80 mV (the voltage change is signified by an asterisk and the ‘I’ state after the quench is noted as ‘I*’, Fig. 2b). The lifetime distribution at +80 mV was not affected by this procedure (Fig. 2b) and we obtained a similar unfolding rate of *k*_I*→T_ = 1.05 ± 0.04 s^−1^ (*n* = 98, the experiment was repeated on additional pores to confirm the reproducibility of the observation). This implies that the rate of the transition from I* to T does not depend on how I* is generated. In these experiments we stepped the voltage manually, and the time spent at +120 mV varied from 0.05 to 2 s before the quench was applied. The lifetime at +80 mV was independent of the time the intermediate spent at +120 mV before the quench (*r*^2^ = 0.007, Supplementary Fig. 2). Therefore, the process is memoryless: the transition rate depends neither on the previous conditions nor the time spent under those conditions. This means that the dynamics of the intermediate at low applied potentials can be investigated after stepping from higher potentials.

### The intermediate refolds to the native state at low forces

**Fig. 3:**
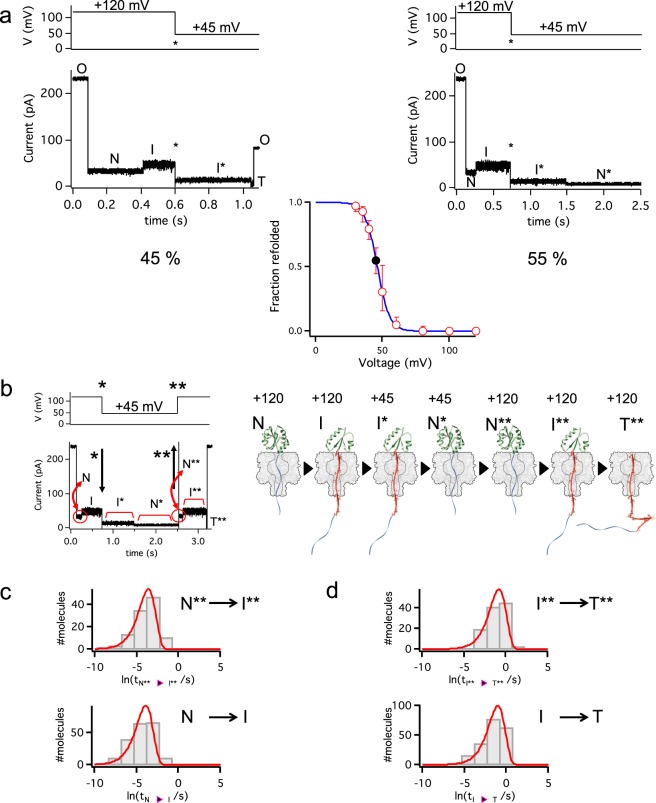
The intermediate I refolds to the native state N at low membrane potentials. **a** The intermediate produced at +120 mV was monitored after a step to +45 mV (asterisk). This state (I*) could either complete unfolding and translocate (left trace) or convert to a low-conductance level that resembled the native state (N*, right trace). At +45 mV, 55% of the molecules (*n* = 92) transitioned to the low-conductance level. The analysis of 524 molecules over the range +30 to +60 mV revealed a sigmoidal dependence of the probability to transition to N* on the applied potential. **b** The newly produced native state (N*) was re-exposed to +120 mV (double asterisk) and the co-translocational unfolding of N** was recorded. **c** The lifetimes of the N and N** states showed similar distributions and were fitted to exponentials, yielding *k*_N→I_ = 24 ± 4 s^−1^ (*n* = 115) and *k*_N**→I**_ = 37 ± 19 s^−1^ (*n* = 115), suggesting that both N and N** were natively folded proteins. **d** The lifetimes of the I and I** states show similar distributions and were fitted to exponentials yielding *k*_I→T_ = 2.8 ± 0.5 s^−1^ (*n* = 198) and k_I**→T_ = 2.3 ± 0.6 s^−1^ (*n* = 115), suggesting that I and I** have similar stabilities.

There is a linear relationship between the applied potential and the force experienced by the negatively-charged oligonucleotide when it is threaded within the pore^37^. Recent estimates using measurements of ssDNA stretching within the hemolysin pore at several voltages suggest that the applied force is ~10 pN at +160 mV^29^. We take this value with caution and only for illustrative purposes. Further we also assume that the relationship between force and voltage is linear across the narrow range of voltages we use in this study. We extended the quenching experiments to lower potentials to observe the dynamics of the protein at low forces (we use an asterisk (*) to denote this experimental condition). Interestingly, instead of completing unfolding and translocation, some molecules in the intermediate state, at values below +60 mV (~3.75 pN) reverted to a state (N*) with lower conductance similar to the conductance of the native state (Fig. 3a, b). Indeed, the I_res_ values obtained for this N* state in the +50 to +30 mV range were a continuation of the monotonic decrease observed for the I_res_ values of the native state (N) in the +120 to +80 mV range (Supplementary Fig. 3). Similarly, the I_res_ values obtained for I* in the +60 to +30 mV range continued the monotonic decrease observed for I in the +120 to +80 mV range (Supplementary Fig. 3). In addition, the fraction of molecules in the intermediate state I* that transitioned back to the putative native state N*, rather than proceeding to complete unfolding and translocation, showed a sigmoidal dependence on voltage with 118 out of 121 molecules transitioning back at +30 mV (~1.9 pN; Fig. 3c). The transition occurred over a narrow range of forces because at +60 mV (~3.7 pN) the refolding transition was rarely observed (only in 5 out of 99 molecules). Interestingly, a narrow range of forces, from 1 to 2 pN, over which a protein transitions between states has also been reported in experiments with optical tweezers^38^ and with magnetic tweezers^39^, where proteins are stretched by pulling on both ends.

In order to confirm that the putative native state (N*) obtained by reversion from I* was truly the native state, we introduced an extra step in the applied voltage protocol. First, a Trx molecule was unfolded to the intermediate state by pulling with +120 mV. Then the voltage was quenched to +30 mV and once the protein had transitioned back to the putative native state the +120 mV potential was restored; we use a double asterisk (**) to denote this experimental condition (Fig. 3c, d). In all cases (*n* = 115), the characteristic current signal that reveals the unfolding and translocation of a natively folded protein was obtained. Importantly, the kinetics of the N → I and I → T transitions are similar to those of N** → I** and I** → T transitions (*k*_N→I_ = 50 ± 10 s^−1^; *k*_I→T_ = 2.8 ± 0.5 s^−1^ *n* = 198; and *k*_N**→I**_ = 38 ± 19 s^−1^; *k*_I**→T_ = 2.3 ± 0.6 s^−1^ *n* = 115) suggesting that N** represents a natively folded protein. Similar observations were made when the quenching voltages were +35, +40 and +45 mV or when the restored voltage was +100 mV, implying that the process is memoryless. Consistent with this interpretation, no correlation was observed between the lifetimes of any of the states (Supplementary Fig. 4). Together, the observations suggest that at low applied potentials Trx is able to refold from the intermediate state to the native state.

### The folding landscape at the early stages of translocation

**Fig. 4:**
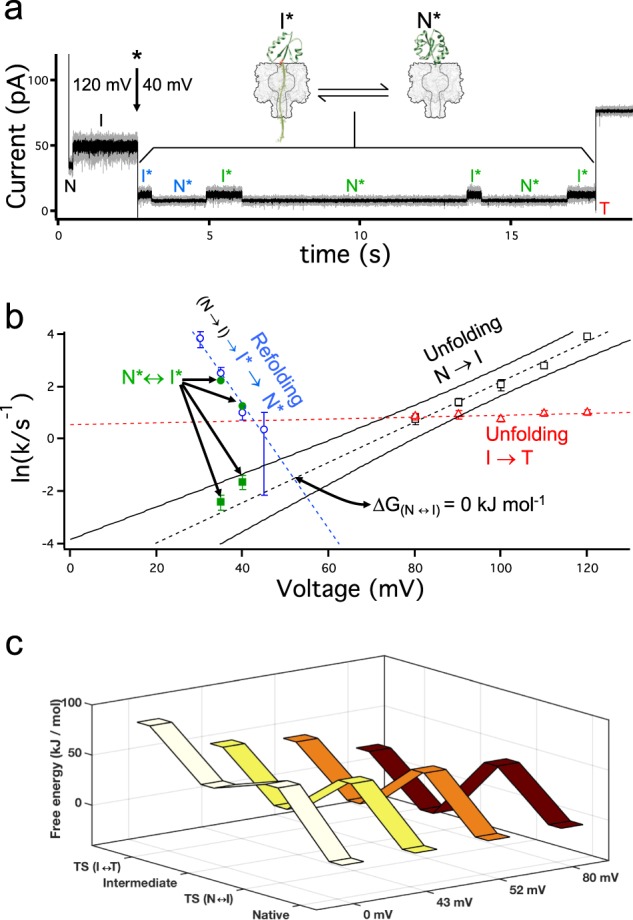
The free-energy landscape at the early stages of co-translocational unfolding. **a** Representative trace of a protein molecule transitioning back and forth between the native (N*) and the intermediate (I*) states at +40 mV (~2.5 pN). Six transitions occurred before the protein completely unfolded and translocated through the pore (T). In gray the original data (sampled at 20 kHz with a low Bessel filter of 5 kHz) and in black the data filtered at 1 kHz. **b** Voltage dependence of the kinetics. The unfilled black squares represent the unfolding kinetics (N → I) in the +80 – +120 mV range. The black dashed line is the best linear fit and the black continuous lines demark the 95% confidence intervals. The green filled squares are the unfolding kinetics (N* → I*) obtained at low voltages on molecules that experienced several N* ↔ I* transitions (203 transitions on 12 molecules at +35 mV and 93 transitions on 23 molecules at +40 mV). The unfilled blue circles represent the refolding kinetics (I* → N*) calculated by using solely the first I* to N* transition as shown in a), The blue dashed line is the best linear fit. The green filled symbols represent the refolding rates calculated from molecules experiencing several N* ↔ I* transitions (203 transitions on 12 molecules at +35 mV and 93 transitions on 23 molecules at +40 mV). **c** Free energy landscape representations at different applied potentials and in the absence of a potential (by extrapolating the rates). The free energies were calculated by taking the native state as zero kJ mol^−1^, and using 10^8^ s^−1^ for the preexponential factor.

When the quenching voltage was kept constant either at +40 or +35 mV, the protein transitioned back-and-forth between the N* and I* states (Fig. 4a). The intermediate was also able to proceed to complete unfolding and translocate through the pore. There were therefore 3 observable steps: the reversible transitions between N* and I*, and the irreversible transition from I* to T. The retrograde release of V5-C109-poly(dC)_40_ into the cis medium was rare at voltages of +30 mV or greater (8 times for 355 molecules).

The refolding time of the intermediate showed an exponential dependence on voltage (Fig. 4b), with rates ranging from *k*_I*→N*_ = 2.7 ± 0.7 s^−1^ at +40 mV (*n* = 89) to *k*_I*→N*_ = 47 ± 14 s^−1^ at +30 mV (*n* = 84) (Supplementary Fig. 5). As expected for a memory-less, reversible process, the refolding rates are similar whether only the initial refolding step after the drop in potential is taken into account or whether the lifetimes of the intermediates from the back-and-forth transitions at low voltages are used (N* ↔ I*, Fig. 4b). Second, the logarithms of the unfolding rates for the native protein at low voltages lies on the extrapolated line of unfolding rates obtained at higher voltages at which unfolding is irreversible (shown in green and black squares respectively in Fig. 4b). Similar to two-end pulling experiments^40,41^, the kinetics are well described by a two-state Markovian process and the voltage-dependent rate constants follow:

$$\begin{array}{l}k_u\left( V \right) = A \cdot e^{\left[ { - \left( {{\mathrm{\Delta }}G_u^\ddagger - V \cdot \alpha \cdot {\mathrm{\Delta }}x_u} \right)/\left( {k_B \cdot T} \right)} \right]}\\ k_f\left( V \right) = A \cdot e^{\left[ { - \left( {{\mathrm{\Delta }}G_f^\ddagger - V \cdot \alpha \cdot {\mathrm{\Delta }}x_f} \right)/\left( {k_B \cdot T} \right)} \right]}\end{array}$$

where *A* is the pre-exponential factor, ${\mathrm{\Delta }}G_u^\ddagger$ and ${\mathrm{\Delta }}G_f^\ddagger$ are the activation free energy barriers for unfolding and refolding respectively, *V* is the applied potential, *α* is the force in pN produced by 1 mV (taken as 0.0625 pN mV^−1^)^29^, ${\mathrm{\Delta }}x_u$ and ${\mathrm{\Delta }}x_f$ are the end-to-end distance changes between N and I in the unfolding direction and in the refolding direction respectively, $k_B$ is the Boltzmann’s constant and T is the absolute temperature. The exponential dependence confirms that the work derived by the movement of charge in the applied potential linearly decreases the free energy barrier. Also, as in two-end pulling experiments^40^, the transition state is closer to the native state as revealed by the voltage dependence of the refolding reaction, which is steeper than that of the unfolding reaction $\left( {{\mathrm{\Delta }}x_u <\, {\mathrm{\Delta }}x_f} \right)$. This closeness to the native state implies that at the transition state the oligonucleotide is threading the barrel and is consistent with the sensitivity of the refolding reaction to the applied voltage. This is relevant because the refolding opposing forces arising from the oligonucleotide re-entry^42^ do not add up to rate limit the refolding reaction. Finally, the complete unfolding of the intermediate is irreversible because the unfolded polypeptide translocates through the pore (red, Fig. 4b).

This allowed us to portray the free energy landscape of the early steps of co-translocational protein unfolding as a function of voltage (Fig. 4c). At potentials above +60 mV, the protein unfolds irreversibly because the refolding barrier is too high. At + 52 mV, unfolding of the native protein and refolding of the intermediate encounter similar free-energy barriers but the intermediate completely unfolds (to T) more readily than it refolds. By contrast, at a potential of +43 mV, which corresponds to a force of ~2.75 pN, the protein has the same probability of proceeding to complete unfolding and of refolding to the native state. The value of the associated potential can be obtained either by extrapolation of the rates (Fig. 4b) or directly from the fraction of molecules observed to refold from the intermediate (Fig. 3a). Both give a similar value of the applied potential (+43 and +46 mV, respectively). Below this voltage threshold, the native state is favored.

### The effect of ligand binding

**Fig. 5:**
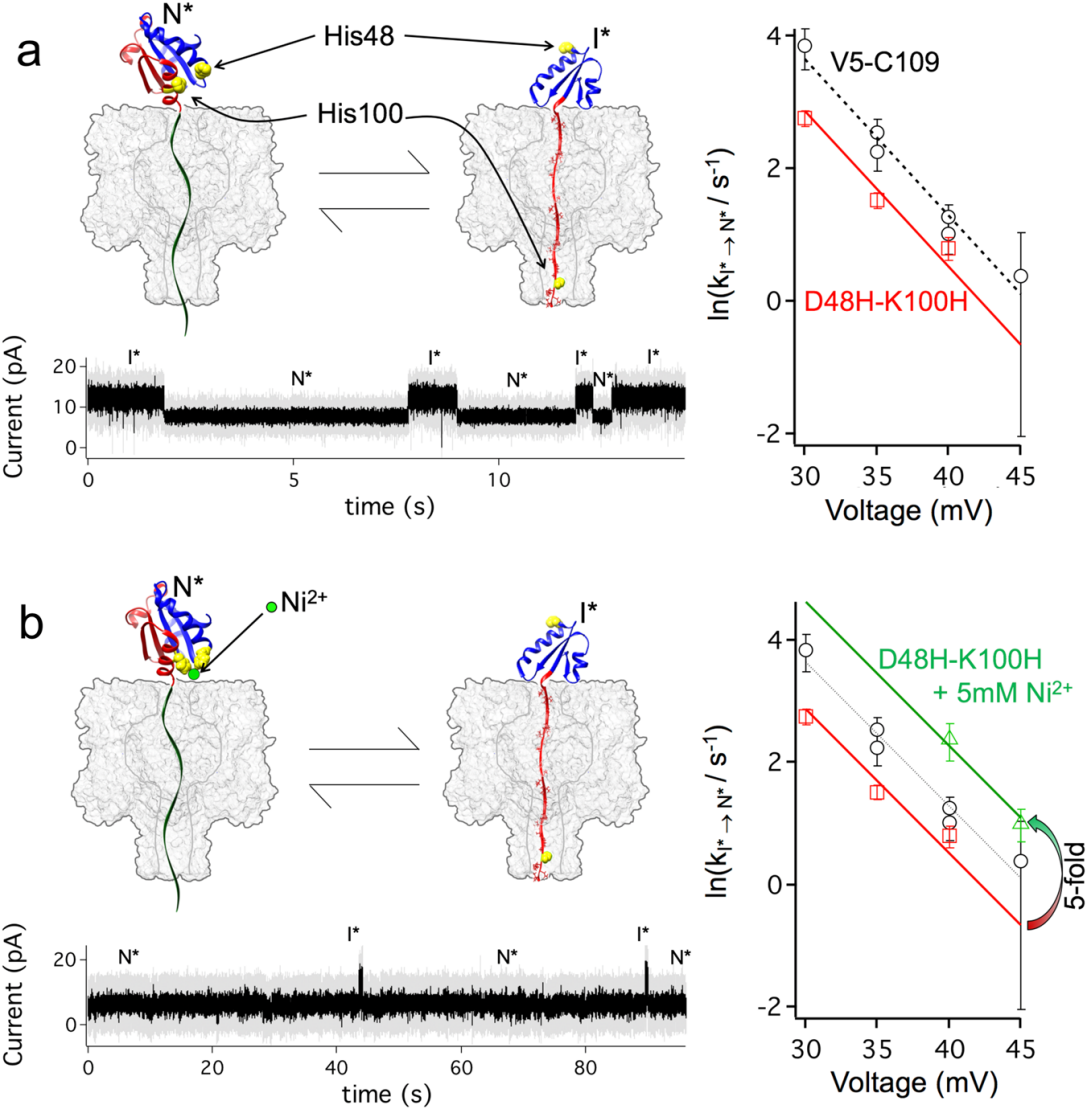
Effect of ligand binding on co-translocational unfolding and refolding. **a** A two-histidine mutant was designed to replace a salt-bridge between Asp-48 and Lys-100 in Trx. The salt bridge present in the native state (N*) is broken in the intermediate (I*)^28^. This mutant fluctuated between the N* and I* states at +40 mV (gray, raw data; black, signal filtered at 1 kHz). Right: dependence of the refolding kinetics on the applied potential. The two-histidine mutant (red) showed an ~2-fold decrease in the refolding rate compared to Trx-V5-C109 (black). **b** When 5 mM Ni^2+^ was added, the two histidine side-chains coordinated the cation altering the kinetics (+40 mV: gray, raw data; black, signal filtered at 1 kHz). Right: dependence of the refolding kinetics on the applied potential. In the presence of 5 mM Ni^2+^, the refolding rate (green) increased ~5-fold, demonstrating an interaction between residues 48 and 100 in the transition state for refolding of the intermediate (I*). The straight lines in **a** and **b** all have the same slope (obtained by a global best-fit to first order polynomials).

We next designed a conditional mutant by introducing a two-histidine metal ion binding site^43^ between the C-terminal α helix and a structured part of the intermediate I (Fig. 5a). We mutated Lys-100 and Asp-48 to histidine (in the V5-C109 background), because these two residues are close to each other in the native state of wild-type Trx and indeed form a salt bridge. In the absence of divalent cations, the residues in the mutant should not interact, but in the presence of Ni^2+^ the two histidine side chains were expected to coordinate the divalent ion (Fig. 5b), mimicking the salt bridge in the non-mutated protein.

In the absence of divalent ions (1 mM EDTA), the native state of the D48H-K100H mutant unfolded to the intermediate state ~20-fold faster than V5-C109 (970 ± 20 s^−1^ at +120 mV, Supplementary Fig. 6). When the voltage was quenched during the appearance of the intermediate, the protein refolded at potentials between +40 and +30 mV. We examined the dynamics at low forces and observed that refolding was only slightly slower than the V5-C109 with rates ranging from *k*_I*→N*_ = 2.2 ± 0.4 s^−1^ at +40 mV (*n* = 260) to *k*_I*→N*_ = 16 ± 2 s^−1^ at +30 mV (*n* = 294) (Fig. 5a and Supplementary Fig. 6). Next, the experiments were repeated in the presence of Ni^2+^. The divalent cation interacts with residues 48 and 100 in the native state, and the effect on the folding and unfolding rates reports on the extent of the interaction in the transition state. In the presence of 5 mM Ni^2+^, the mutant was slightly more mechanically stable in the native state than V5-C109 as judged by the slower unfolding at +120 mV (*k*_N*→I*_ = 16 ± 1 s^−1^, Supplementary Fig. 6). Refolding at +40 mV, on the other hand, was 5-fold faster than in the absence of Ni^2+^ (*k*_I*→N*_ = 11 ± 3 s^−1^, Fig. 5b). Surprisingly, in the presence of 5 mM Ni^2+^, refolding is also faster than the V5-C109, which we interpret as the interaction between His-48 and His-100 in the presence of Ni^2+^ being stronger in the mutant than the ion pair in V5. Taken together, the results suggest that in the transition state, the interaction between residues 48 and 100 is at least partially formed and therefore the transition state is native-like^43^. This situation is similar to the transition state for the mechanical unfolding of proteins by 2-end pulling^40^, where the end-to-end distance in the transition state is typically less than 1 nm away from that in the native state.

## Discussion

Our results yield the free energy required to produce the first threaded translocation intermediate by using:

$${\mathrm{\Delta }}G_{N \to I}\left( V \right) = k_B \cdot {\mathrm{T}} \cdot N_A \cdot \left( {\ln k_{N \to I}\left( V \right) - \ln k_{I \to N}\left( V \right)} \right)$$

with *k*_*B*_ the Boltzmann’s constant, T the absolute temperature and *N*_*A*_ the Avogadro’s constant. By extrapolation of the rates, we obtained ΔG_N→I_ = 38 ± 8 kJ mol^−1^ [95% C.I.] in the absence of a membrane potential, consistent with the experimentally determined free energy for chemically- or temperature-induced unfolding, 35 and 32 kJ mol^−1^ respectively^44,45^. This implies that the free energy change to obtain the intermediate is about the same as the overall free energy to unfold the protein. The free energy difference between the native and the threaded intermediate state varies dramatically with the application of force and becomes zero at an applied membrane potential of +52 mV, which produces a force of ~3.1 pN.

In order to contextualize our results, several factors should be considered. In the α-HL pore, most of the membrane potential drops across the transmembrane barrel^46,47^ (roughly ~4 nm long, ~1.3 nm wide), and the ~10 negative charges on the phosphodiester bonds of the leader oligo, which are accommodated within the barrel^48^, sense the field producing the pulling force that drives unfolding. By comparison, during protein import into mitochondria and by the bacterial SecYEG translocon, the associated membrane potentials have been estimated to contribute forces close to those generated in our work (6 to 12 pN)^20,22^. Indeed, both systems feature transmembrane pores of similar diameter and length to those of α-HL^9,11^. The membrane potential across the mitochondrial inner membrane is around −180 mV^18^ and the periplasm and ER, around −100 and −74 mV, respectively^49,50^. In this study, we applied voltages one-half to one-third of these values, but because the density of charges in natural protein substrates is also a half^51^ or a third^4^ of the charge density in our model system we end up applying similar forces. The small differences in the estimated forces/voltages may arise because force is estimated in the αHL pore using data for DNA stretching at different voltages^29^ and in bacteria, mitochondria or the ER by using voltage- or ion-sensitive dyes^49,50^. In addition, our approach does not take into account other possible mechanisms that can contribute to the unfolding and translocation of polypeptides, such as biased diffusion^52^.

The rates observed in our model system match those recently reported for bacterial post-translational translocation through the inner membrane by the Sec machinery^6^. In this system there is a hidden stage, once the substrate has engaged the open translocon and before the substrate is actively translocated, that lasts for roughly 5 s in the absence of the chaperone Sec B. This phase, which is independent of substrate length, is rate-limiting in the translocation of small substrates and is likely associated with protein unfolding, because the presence of the chaperone catalyzes unfolding, both reducing ATP consumption and accelerating the process^6^. Our unfolding kinetics at low voltages, although for a different substrate, are consistent with this timescale. We also observe that refolding of the substrate pulls from the threaded polypeptide out of the pore. This may be the mechanism of protein release from translocons as it is for nascent chain release from the ribosome once translation is finished^53^.

We therefore suggest that the biophysics underlying our model system may be generalizable to the natural systems discussed here, because in all of them a protein substrate is pulled from one terminus through a nanometer-diameter pore. The model system may be useful for exploring several issues: for example, the role of the residues located at the entrance of the pore, which may facilitate substrate unfolding or prevent retrograde movement of threaded intermediates, or the differences between pores formed from β barrels and α helices with regard to co-translocational unfolding. Answers to these questions may assist in the development of single-molecule protein analysis with nanopores^36^.

## Methods

### Preparation of thioredoxin mutants

A pET 20+ plasmid carrying the *thioredoxin 2* gene from E. coli including the mutations A22P-I23V-C32S-C35S-P68A-C109 (V5-C109) was transformed into E. coli BL21 (DE3) cells and grown for 16 h at 37 °C with shaking in 0.5 L of LB medium supplemented with 10 mg L^−1^ of kanamycin. Cells were collected by centrifugation (10,000 *g*, 10 min) and suspended in fresh medium with kanamycin. After 1 h at 37 °C with shaking, expression was induced with isopropyl β-D1-thiogalactopyranoside (IPTG, 0.4 mM). After 4 h, the cells were collected by centrifugation (10,000 *g*, 10 min) and suspended in 25 mL of buffer containing 30 mM Tris·HCl, 1 mM EDTA, 10 mM DTT, pH 8.3 (buffer A). The cells were placed on ice and lysed by sonication. The tip of the sonicator was immersed in the cell suspension and five 45 s bursts of sonication were applied, each followed by a 45 s break.The resulting suspension was centrifuged (10,000 *g*, 10 min) to remove the cell debris. The supernatant was separated on a Superdex 75 16/60 column (GE Healthcare) and the peak containing the protein was loaded onto a 1 mL monoQ column (GE Healthcare) equilibrated in buffer A. The protein was eluted with a linear gradient from 0 to 1 M NaCl (in the same buffer). The fractions that contained the protein were stored at −20 °C. The mutant D48H-K100H in the V5-C109 background was prepared by site-directed mutagenesis (QuikChange II, Agilent) and purified in the same manner.

### Preparation of thioredoxin-poly(dC)_40_ constructs

The poly(dC)_40_ oligonucleotide with a 5′ C6 amine activated with SMCC-maleimide (HPLC grade) was obtained from Biomers. The lyophilized oligonucleotide was resuspended in 10 mM HEPES, pH 7.0 (0.15 mg, 1 mg/mL). The thioredoxin was exchanged into the same buffer with a PD10 column ensuring that all the DTT was removed and 2 mg of the protein (2 mg/mL) was mixed with the oligonucleotide and incubated for 2 h at room temperature. The mixture was purified with a monoQ column (GE Healthcare) and a linear gradient from 0 to 1 M KCl in the same buffer. The V5-C109-oligonucleotide complex was pure as judged from a single band in SDS-PAGE and the concentration estimated using the molar extinction coefficient for the oligonucleotide at 260 nm. The purified conjugate was stored at −20 °C.

### Preparation of hemolysin heptamers

α-HL monomers were purified from BL21(DE3) cells (Novagen) transformed with a pT7 plasmid containing the Staphylococcus aureus *wild-type α-HL* gene. Overexpression was induced with 1 mM IPTG at 25 °C for 4 h. Cells were collected by centrifugation and suspended in purification buffer (30 mM Tris·HCl, 50 mM EDTA, pH 8.0). The cells were lysed by sonication and the soluble fraction was obtained by centrifugation at 100,000 *g* for 45 min. α-HL monomers were precipitated with 75% ammonium sulfate and recovered by centrifugation at 40,000 *g* for 50 min. The precipitate was resuspended in 5 mL of 10 mM sodium acetate, pH 5.0, containing 20 mM NaCl, and dialyzed against the same buffer overnight at 4 °C. The suspension was centrifuged (40,000 *g* for 50 min) and the supernatant was separated on a Superdex 200 16/60 column (GE Healthcare). The peak containing the monomers was identified by SDS-PAGE and stored at −20 °C without further purification.

To produce heptamers, the monomers were incubated with erythrocyte membranes prepared from fresh rabbit blood. Briefly, 100 mL of blood was collected and mixed with 50 mL of citrate anticoagulant (80 mM citrate, 140 mM dextrose, pH 7.4) and the red blood cell fraction obtained by centrifugation (500 *g*, 10 min) and washed with 150 mM NaCl, 10 mM sodium phosphate, pH 7.4, three times at 4 °C. Lysis was produced by 1:1000 dilution, drop by drop, of the erythrocytes into hypotonic solution (5 mM Hepes, pH 7.4) at 4 °C with stirring. The erythrocyte membranes were isolated by centrifugation at 20,000 *g* for 20 min at 4 °C and washed with the same buffer three times. To remove the cytoskeleton, the membranes were diluted in 2 M NaCl, 10 mM Hepes, pH 7.4, and centrifuged at 20,000 *g* at 4 °C for 20 min, three times. The final pellet was suspended in the same buffer and stored at −20 °C (the concentration of membranes was not determined). Heptamers were obtained by incubating 50 μL of membranes with 250 μL of α-HL monomers (0.3 mg mL^−1^) for 5 min. The membranes were recovered by centrifugation (20,000 *g*, 5 min) and washed 3 times with 2 M sodium chloride, 10 mM Hepes, pH 7.4. The heptamers were extracted by incubation of the membranes with 45 μL of buffer supplemented with 5 μL of 10% (w/v) sodium dodecyl sulfate for 20 min. The heptamers were collected from the supernatant after 20 min centrifugation at 20,000 *g* and stored at −80 °C.

### Single-molecule nanopore measurements

We carried out single-molecule electrical recordings in planar lipid bilayers made of 1,2-diphytanoyl-sn-glycero-3-phophocholine (Avanti Polar Lipids) by the Montal and Mueller method across an aperture, 50-100 μm in diameter, in a 25-μm-thick Teflon film (Goodfellow) that had been painted with 1% hexadecane in pentane^31^. The film separated two compartments (*cis* and *trans*), which were connected to the headstage of an amplifier Axopatch 200B (Molecular Devices) with Ag/AgCl electrodes, *cis* grounded. The amplifier was connected to a digitizer (Digidata 1440 A, Molecular Devices). A PC was used to collect data and control the amplifier with Clampex 10.5 software (Molecular Devices). Both the *cis* and *trans* compartments were filled with 2 M KCl, 10 mM Hepes, pH 7.2, at 22 ± 1.5 °C. When required, the *cis* compartment was supplemented with 5 mM nickel sulfate or 1 mM EDTA (final concentrations). After formation of a bilayer, 1 μL of heptamerized α-HL was added to the *cis* compartment. Following the first insertion of a pore, the remaining heptamerized α-HL was removed by perfusion with 10 chamber volumes of recording buffer. The signal was filtered with a low-pass Bessel filter of 5 kHz and data were collected at a sampling rate of 20 kHz.

### Statistics and reproducibility

Each experimental condition was assayed on at least 3 independent experiments. Raw data were analyzed with ClampFit (Version 10.6.0.13) to obtain the IRES (I_RES%_, the ionic current level as a percentage of the current level of the open pore) and event dwell times. Further data analysis was carried out with Igor Pro (Wavemetrics) and MatLab. Rate estimates and their confidence intervals were obtained by bootstrap: dwell-time data were resampled with replacement to construct 1000 data sets of equal size to the original data set. For each of these we constructed a histogram with time on a natural logarithm scale. Each histogram was fitted to the exponential distribution:

$$f\left( x \right) = {\rm{A}} \cdot \hat k \cdot e^{\left( {x - \hat k \cdot e^x} \right)}$$

where $\hat k$ is the rate and *x* is time. This provided 1000 estimations which were fitted to a normal distribution to obtain the mean (the estimator of $\hat k$) and the 95% confidence interval in the rate parameter estimation (1.96 times the standard deviation of the distribution).

### Reporting summary

Further information on research design is available in the Nature Research Reporting Summary linked to this article.

## Supplementary information

Suppemental Information

Reporting Summary

## Data Availability

Dwell times are stored in hard drive. Relevant data and/or materials are available upon reasonable request from D. R.L.
