# Supplementary material for: Free-energy landscapes of membrane co-translocational protein unfolding
Source: Commun Biol. 2020 Apr 3;3:160. doi: 10.1038/s42003-020-0841-4 (PMC7125183; doi:10.1038/s42003-020-0841-4)
Supplement: Supplementary file 1 — Suppemental Information [file 42003_2020_841_MOESM1_ESM.pdf]

Supplementary Figure 1

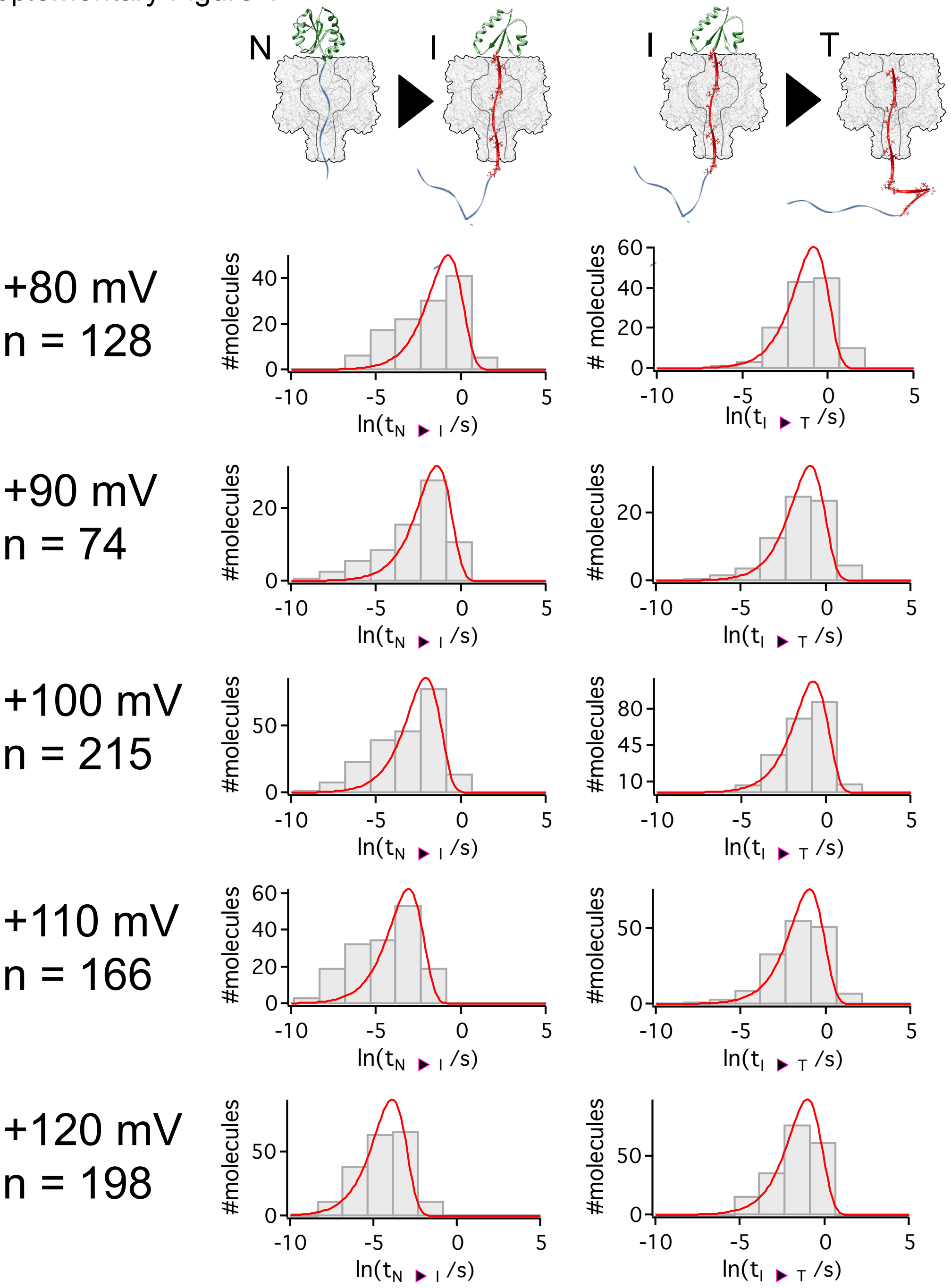

Supplementary Figure 1. Co-translocational unfolding kinetics of V5-C109-poly(dC)$_{40}$. Top, co-translocational unfolding occurs in two steps. In a first step, C-terminal part of the protein (to which the oligonucleotide is attached) separates from the N-terminal region to produce an intermediate (I). In I, the C terminus has threaded into the pore and an N-terminal folded domain lies outside the pore. In a second step, I fully unfolds, allowing completion of translocation by diffusion of the polypeptide chain through the pore. Below, dwell-time histograms (natural logarithmic scale) at different voltages for N→I and I→T fitted to single-exponential distributions. The numbers of single-molecule observations (n) used to construct each histogram is shown.

Supplementary Figure 2

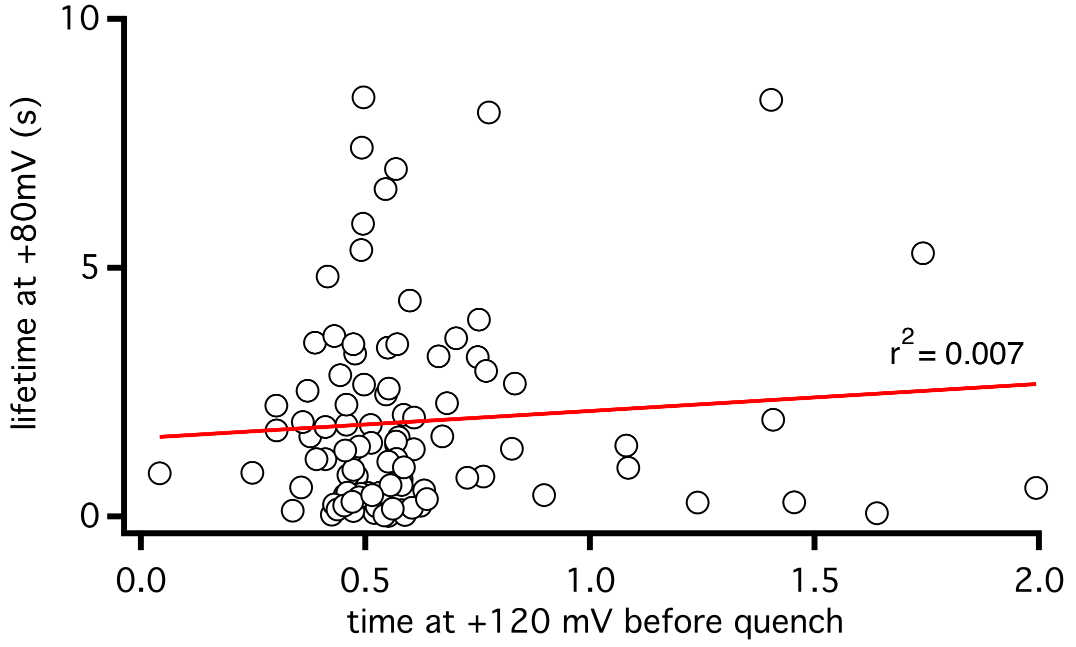

Supplementary Figure 2. The intermediate is memory-less. 98 molecules of V5-C109-poly(dC)$_{40}$ were pulled into the pore at an applied potential of +120 mV until the native state produced the intermediate state. During the intermediate state and after an arbitrary time, the potential was stepped to +80 mV. There is no correlation between the time the intermediate spent at +120 mV (before the voltage step) and the time it took to fully unfold the intermediate at +80 mV, a characteristic of a memory-less process.

Supplementary Figure 3

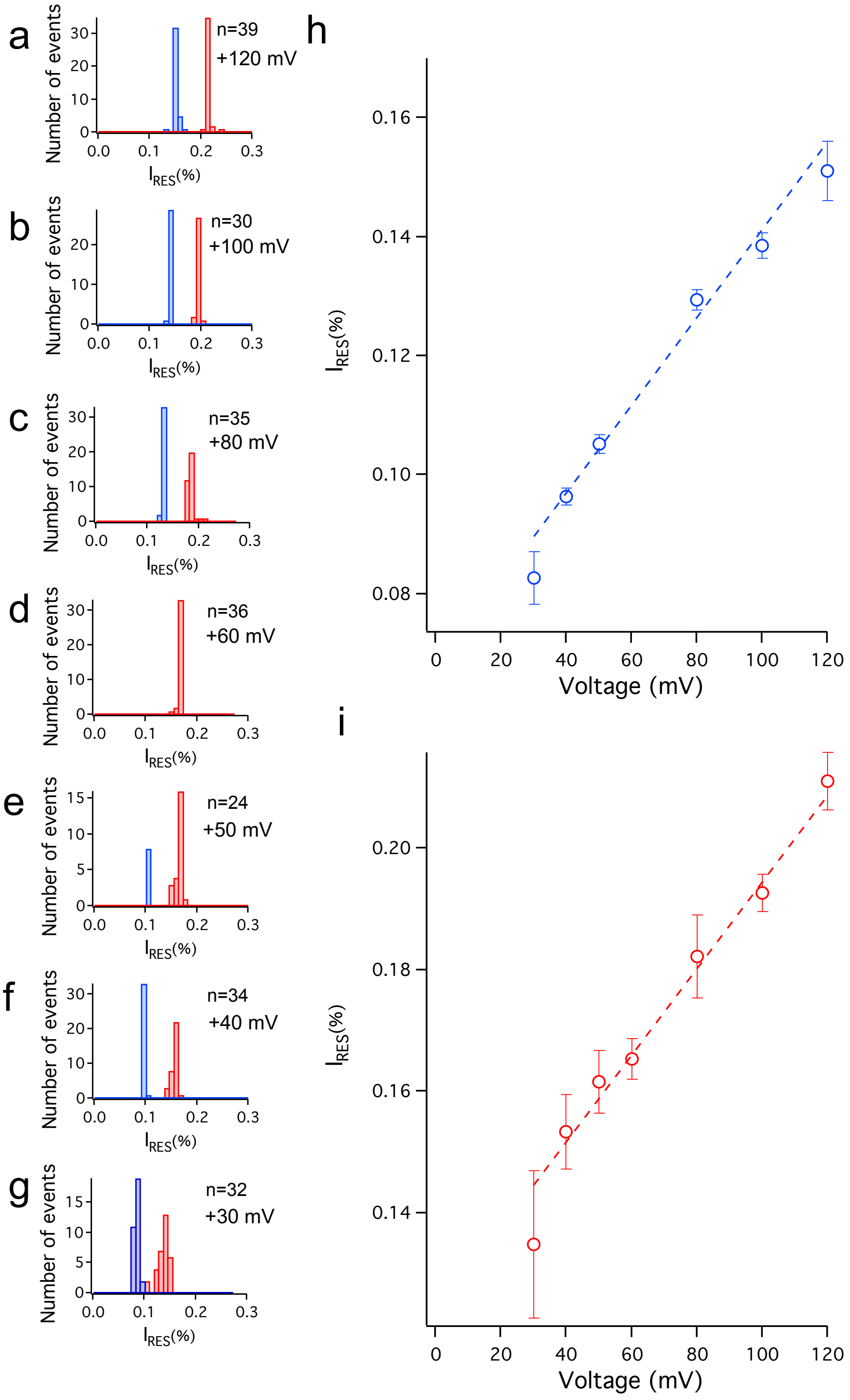

Supplementary Figure 3. Residual currents of the native (N) and intermediate (I) states. a) to **g)** Residual current histograms ($I_{RES(\%)}$) at +120, +100, +80, +60, +50, +40 and +30 mV respectively, with the native state (N) in blue and the intermediate state (I) in red. **h)** Voltage dependence of the residual current produced by the native state (N, error bars are standard deviations). **i)** Voltage dependence of the residual current produced by the intermediate (I). The dashed lines are linear fits. All data were collected with the same αHL pore to avoid pore-to-pore variation.

Supplementary Figure 4

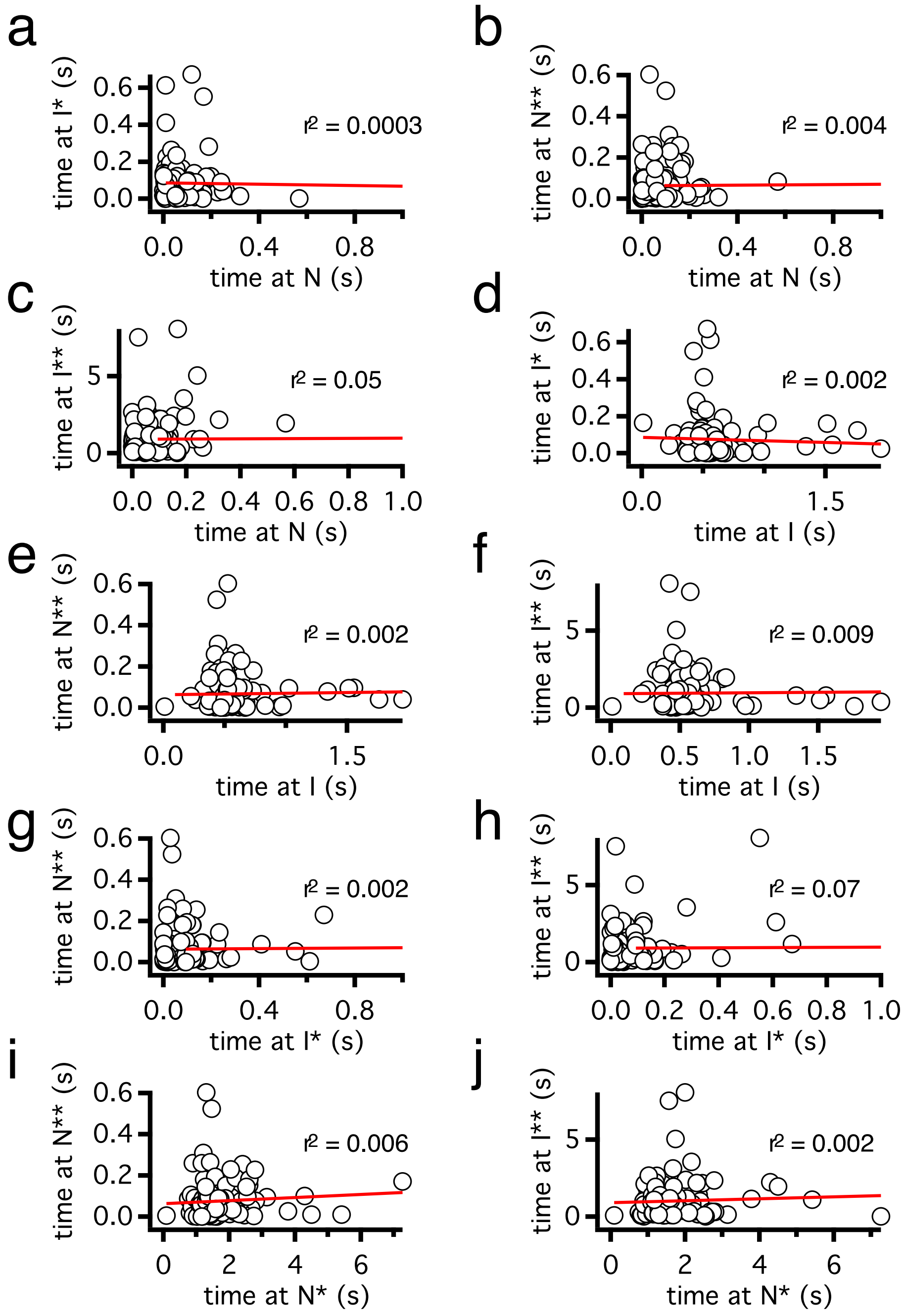

Supplementary Figure 4. The refolding of the intermediate (I) is a memory-less process. **a)** to **j)**, Plots of dwell times obtained in 115 molecules. For each one, V5-C109-poly(dC)$_{40}$ was captured and unfolded to produce the intermediate at +120 mV (I). After an arbitrary delay, the voltage was quenched to +30 mV (I*). The intermediate at low voltage (I*) refolded to produce again the native state (N*). After an arbitrary time, the potential of +120 mV was restored and the native state (N**) unfolded to produce an intermediate (I**) that completed unfolding and translocation (see Figure 3b in the main text for details). The time spent any given state does not affect the time spent at any other state as shown by the lack of correlations (red, a best linear fits, with values of r^2); all combinations shown.

Supplementary Figure 5

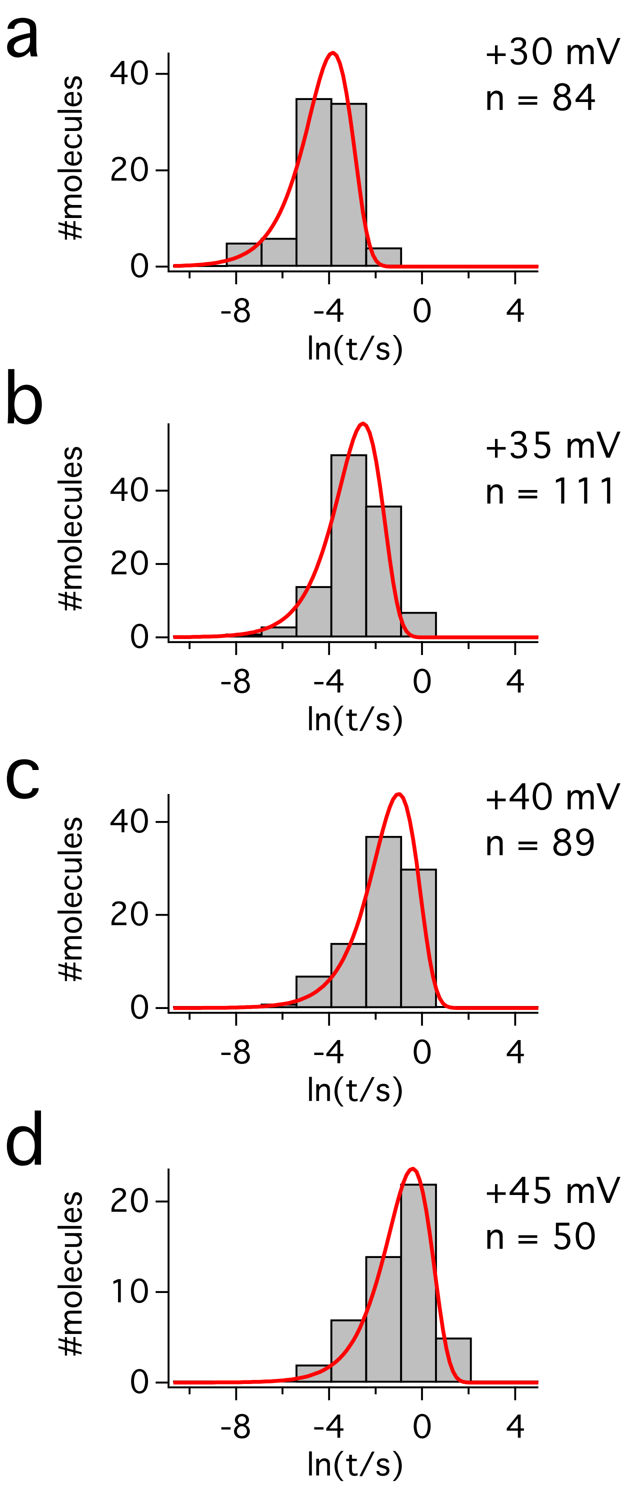

Supplementary Figure 5. The refolding kinetics are voltage dependent. **a)** to **d)** event histograms showing the dwell times of the intermediate (I*) at different applied potential. The red lines are the best fits to exponential distributions which gave rates $k_{I^*\rightarrow N^*}$(30mV)= 38 [66 - 10] s^{-1}; $k_{I^*\rightarrow N^*}$(35mV)= 8.6 [15 – 2.2] s^{-1}; $k_{I^*\rightarrow N^*}$(40mV)= 2.4 [4.2 – 0.6] s^{-1}; $k_{I^*\rightarrow N^*}$(45mV)= 1.4 [2.4 – 0.4] s^{-1}. Data was collected in at least three different pores. The values in brackets are the 95% C.I.

Supplementary Figure 6.

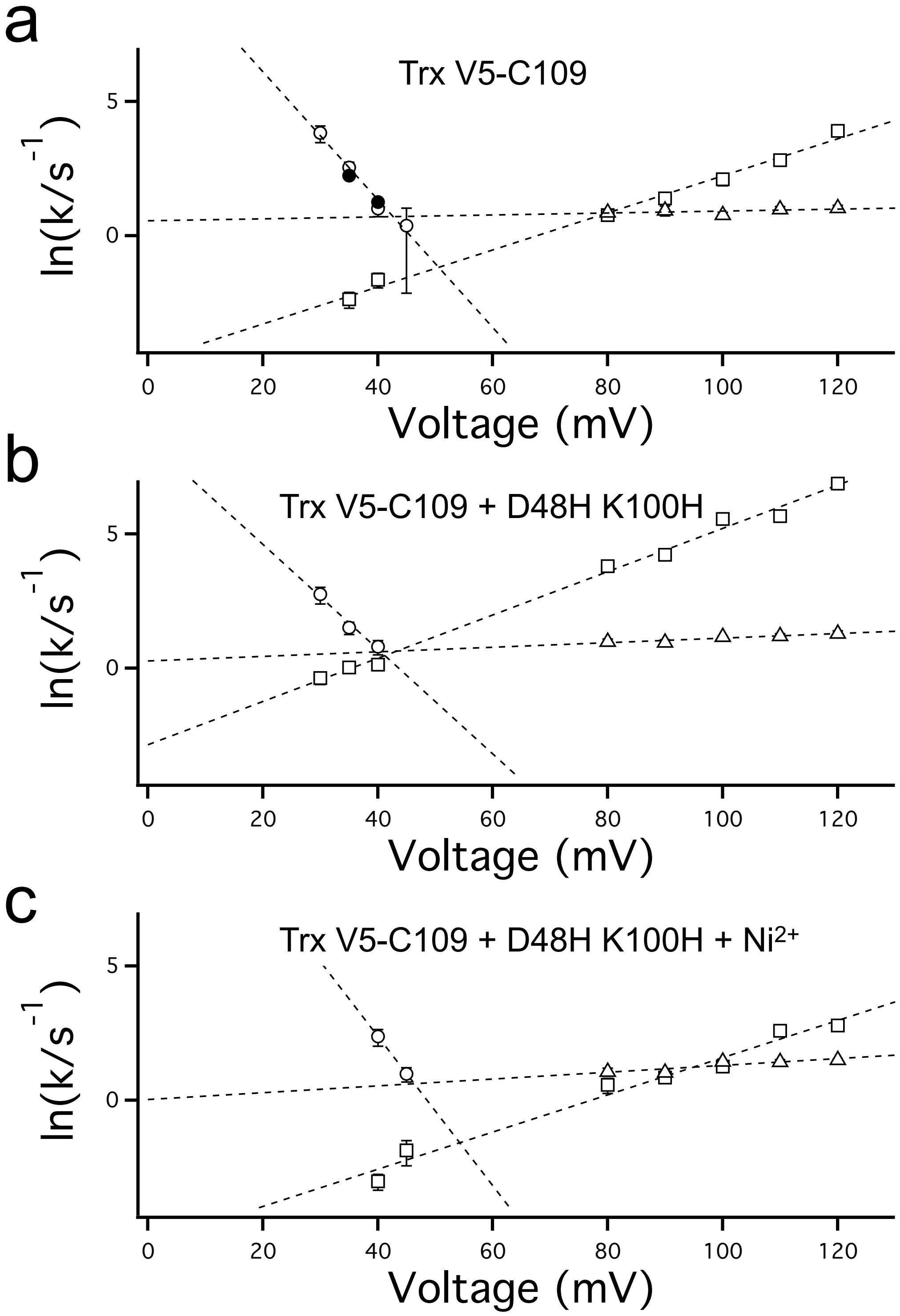

Supplementary Figure 6. Kinetics of unfolding and refolding. **a)** V5-C109-poly(dC)$_{40}$: ▢, rate constant for N→I; o, rate constant for I→N (filled when obtained from multiple N* ↔ I* transitions); △, rate constant for I→T. **b)** V5-C109-poly(dC)$_{40}$ with D48H-K100H mutations in the absence of divalent ions: ▢, rate constant for N→I; o, rate constant for I→N; △, rate constant for I→T . **c)**

V5-C109-poly(dC)$_{40}$ with D48H-K100H mutations in the presence of 5 mM Ni^{2+}: ▢, rate constant for N→I; o, rate constant for I→N; △, rate constant for I→T Lines show best linear fits. Each data point was derived from experiments carried out with 3 or more different αHL pores.
